# Microstructural Characterization of Additively Manufactured AISI 4140 Parts Using Magnetic Barkhausen Noise

**DOI:** 10.3390/ma19183858

**Published:** 2026-09-10

**Authors:** Christian Krämer, Volker Schulze, Stefan Dietrich

**Affiliations:** Institute for Applied Materials, Karlsruhe Institute of Technology, 76131 Karlsruhe, Germany; volker.schulze@kit.edu (V.S.); stefan.dietrich@kit.edu (S.D.)

**Keywords:** magnetic barkhausen noise, additive manufacturing, laser beam powder bed fusion, AISI 4140, residual stress, non-destructive testing

## Abstract

This study investigates the application of magnetic Barkhausen noise (MBN) measurements for assessing the microstructural and residual stress characteristics of additively manufactured AISI 4140 steel. The research explores the influence of part geometry, residual stresses, and material hardness on the MBN signal, particularly in thin-walled structures produced using laser-based powder bed fusion (PBF-LB). Results indicate a strong correlation between wall thickness and MBN intensity, attributed to the penetration depth of the magnetic field and the resulting amplification in thinner sections. Furthermore, an anisotropic MBN response is observed due to residual stress distributions, with tensile stresses leading to a higher Barkhausen signal. These findings highlight the potential of MBN as a non-destructive evaluation method for quality assessment in additively manufactured components supporting future approaches for non-destructive assessment of material conditions in additively manufactured components.

## 1. Introduction

Additive manufacturing (AM) has become an increasingly important manufacturing technology due to its ability to produce complex geometries and highly individualized, load-adapted components. Among the available processes, powder-based techniques are particularly relevant for metallic materials. One of the most widely used approaches is laser beam powder bed fusion (PBF-LB), which is based on the local melting and consolidation of metal powder [1].

The highly localized heat input and rapid solidification inherent to the PBF-LB process result in a characteristic microstructure that differs significantly from conventionally manufactured materials. Due to the layer-wise manufacturing strategy and the repeated remelting of previously deposited material, additively manufactured components experience a complex thermal history. The geometry of the melt pools, which typically extend over several layers and hatch distances, leads to the formation of a cellular microstructure. These cells are separated by the boundaries of former melt pools, where altered cooling conditions result in locally different microstructural features. Furthermore, elongated grains commonly develop along the build direction, causing pronounced anisotropy in the material properties [1]. These characteristic microstructural features and their dependence on processing conditions have been extensively reported for a variety of additively manufactured steels [2,3]. However, studies specifically addressing additively manufactured quenching and tempering (Q&T) steels remain comparatively limited. For AISI 4140, Wang et al. reported a heterogeneous microstructure consisting of fresh martensite, bainite, tempered martensite, and carbides. In addition, the microstructure was found to be considerably finer than that of conventionally manufactured material [4]. Schüßler et al. investigated the influence of base plate preheating on the microstructure of AISI 4140 [5].

Besides determining the microstructure, the thermal history during the PBF-LB process strongly influences the residual stress state. The localized heat input generates steep temperature gradients, which lead to the formation of residual stresses during cooling. In Q&T steels, this effect is further intensified by solid-state phase transformations occurring during repeated heating and cooling cycles. As a result, the material experiences multiple remelting and tempering processes before reaching its final condition. Typical residual stress distributions in additively manufactured components exhibit a stress-relieved zone at the surface, tensile residual stresses close to the surface, and compressive stresses within the component interior [6]. Consequently, both the microstructure and the residual stress state are closely linked to the local thermal conditions during manufacturing.

The strong dependence of material properties on local process conditions presents a major challenge for the qualification and design of additively manufactured components. To ensure sufficient reliability, AM parts are often designed conservatively, which limits the economic and ecological benefits of the technology. Non-destructive testing (NDT) therefore offers significant potential, as it enables the assessment of local material conditions without destructive investigations and may support a more efficient utilization of the material.

**Figure 1:**
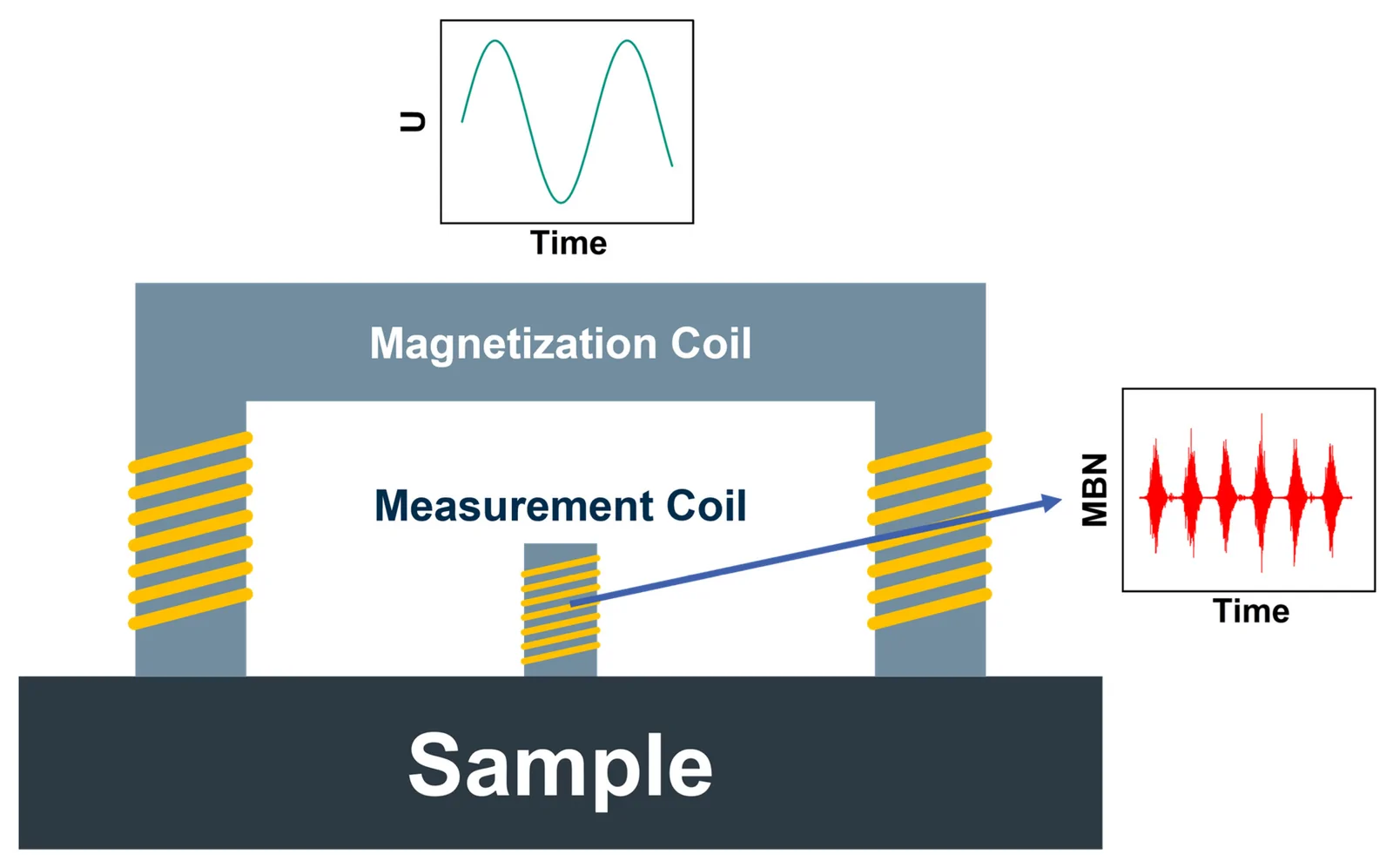
Schematic illustration of the Barkhausen measurement setup.

Among the available NDT methods, magnetic Barkhausen noise (MBN) is particularly promising for ferromagnetic steels because it is sensitive to both microstructural features and residual stresses. MBN is generated during magnetization of a ferromagnetic material. A schematic illustration of the measurement setup is shown in Figure 1. Ferromagnetic materials consist of magnetic domains separated by domain walls, the so called Bloch walls [7]. During magnetization, domains aligned with the external magnetic field grow through the movement of these domain walls. Their motion is impeded by obstacles such as grain boundaries, dislocations, precipitates, and residual stresses. When a pinning obstacle is overcome, a rapid discontinuous movement occurs, generating an electromagnetic pulse that contributes to the Barkhausen signal. Statistical parameters such as the root mean square (RMS) value or the full width at half maximum (FWHM) are commonly used to characterize the measured signal. Among these parameters, the RMS value is most frequently employed due to its high statistical robustness.

In conventional manufacturing, correlations between MBN and residual stresses [8,9,10] as well as microstructural changes [11,12] are well established. Consequently, MBN is widely used for quality control of manufacturing processes involving localized heat input, for example for the detection of grinding burn [13]. Vourna et al. provided a comprehensive review of the applications of MBN for material characterization and compared its suitability with other non-destructive testing methods. They highlighted the sensitivity of MBN to microstructural features and residual stresses, demonstrating its potential for the non-destructive characterization of these material properties [14]. The application of MBN to additively manufactured materials is still comparatively recent. Oliveira et al. investigated the influence of build orientation, residual stresses, and microstructure on the magnetic properties of additively manufactured maraging steels [15,16]. They observed reduced Barkhausen activity in regions with compressive residual stresses and increased signals in areas subjected to tensile stresses. Furthermore, changes in microstructure induced by heat treatment were reflected in the Barkhausen response. For additively manufactured AISI 4140, Engelhardt et al. demonstrated that the process parameters influence micro magnetic properties, with the coercive field strength decreasing at higher volumetric energy densities [17]. Moreover, Kulkarni et Al. reviewed on the influence of residual stresses in additive manufactured metallic parts and also the correlation of the Barkhausen signal and microstructural changes [18]. However, studies investigating the influence of component geometry on the Barkhausen response of additively manufactured AISI 4140 are currently lacking.

Thin-walled structures are of particular interest for many AM applications, especially for heat exchangers, where a reduction in wall thickness can improve the surface-to-volume ratio and thereby increase efficiency [19,20]. At the same time, decreasing wall thickness significantly alters the thermal conditions during manufacturing and therefore affects both the resulting microstructure and residual stress state. Despite the industrial relevance of such structures, no studies have yet examined the Barkhausen response of additively manufactured thin-walled AISI 4140 components and the associated geometry-induced size effects. Therefore, this work investigates the applicability of MBN measurements for the microstructural characterization of additively manufactured AISI 4140 thin walls.

## 2. Materials and Methods

### 2.1. Sample Fabrication

In this study, samples manufactured from quenching and tempering steel AISI 4140 were investigated. The chemical composition of the powder is shown in Table 1. Inductively coupled plasma optical emission spectroscopy (ICP-OES) was used for the measurement of the Mn, Cr, Si, Mo and Fe content. The carbon content was measured using an elemental CS-Analyser. The powder had a particle size fraction of 15–53 µm.

**Figure 2:**
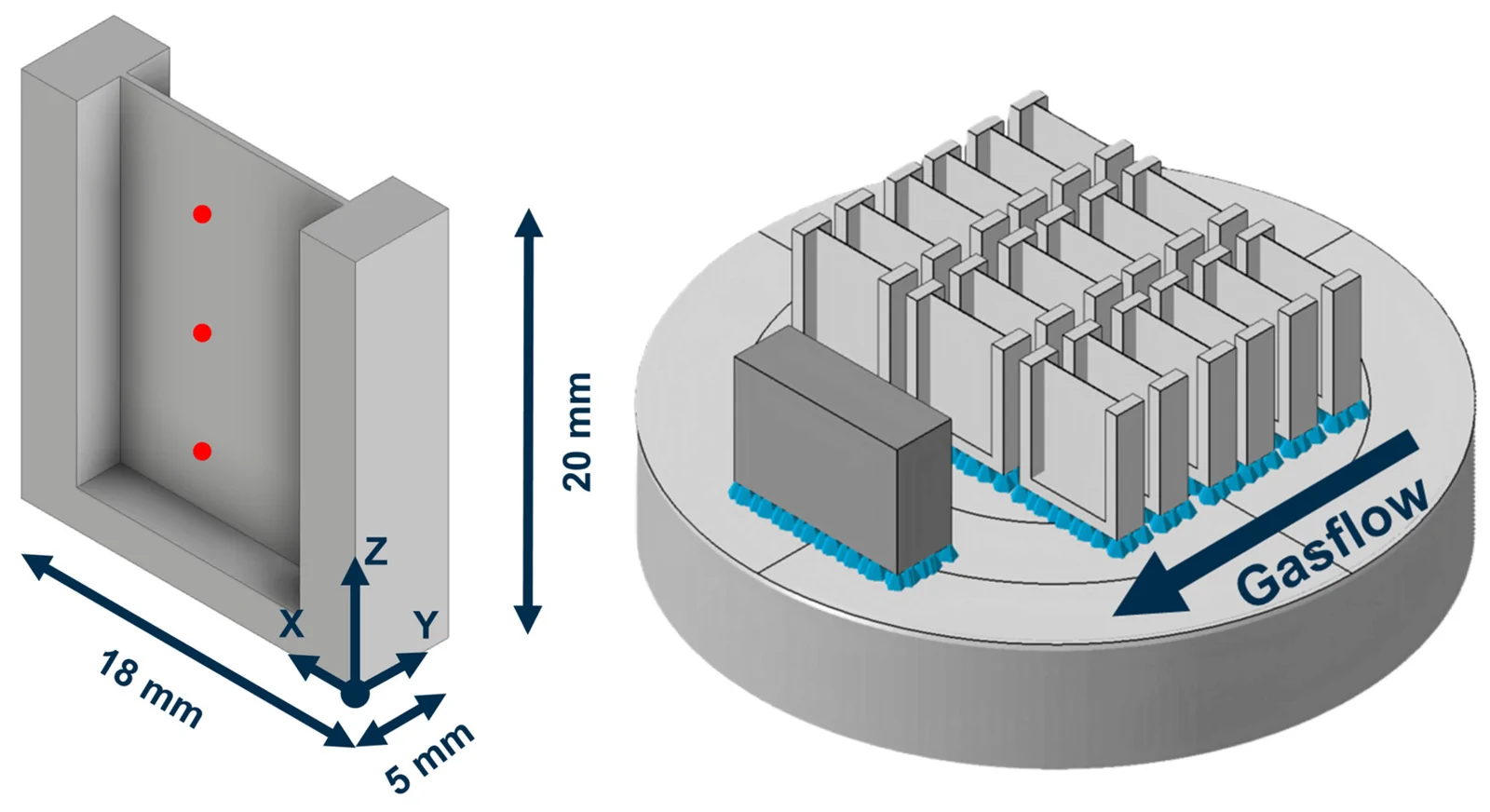
(**Left**) Sample with the measurement points (red). (**Right**) Print setup. The z-direction describes the build direction.

All samples were manufactured using an Orlas Creator PBF-LB system (OR Laser GmbH) equipped with a 250 W Yb fiber laser. The processing parameters are summarized in Table 2. The sample geometry and its orientation relative to the argon inert gas flow are shown in Figure 2. The specimens consist of a U-shaped frame with an internal thin wall.

**Figure 3:**
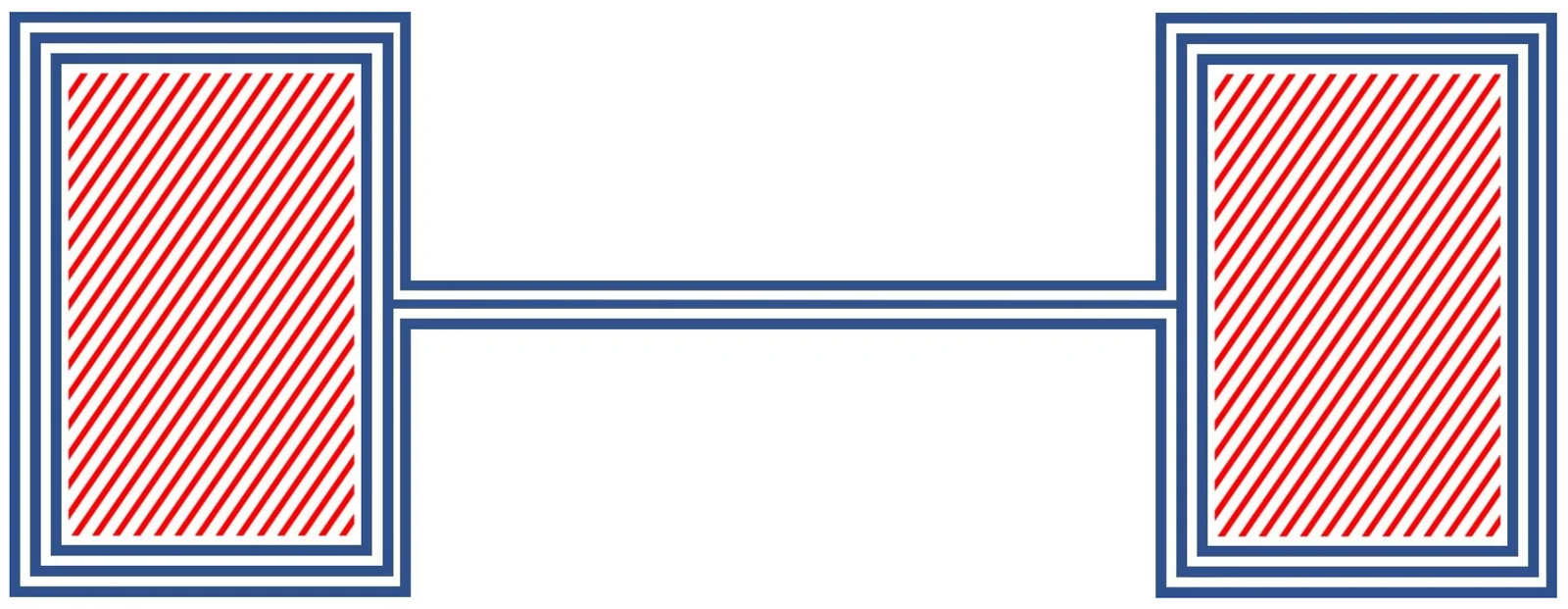
Schematic illustration of the scan strategy. Blue: Contour-laser-paths. Red: Hatch-paths.

Variations in wall thickness were realized by applying 1 to 6 parallel scan lines. The scan strategy for the sample with three laser tracks is illustrated in Figure 3. Contour tracks (blue) were exposed from the outside to the inside. Hatch vectors (red) were rotated by 66° between successive layers. The hatch spacing and number of laser tracks in the hatch region were kept constant.

### 2.2. Magnetic Barkhausen Noise Measurement Setup

The MBN measurements were performed using a Rollscan 350 device from Stresstech GmbH with a general-purpose sensor. This sensor is built according to the schematic illustration in Figure 1 and has a spring-mounted measurement coil, which ensures repeatable measurements by controlling the contact force and reducing systematic error. For each sample, three measurements per direction were performed on both sides of the sample. The two measurement directions were along the build direction (Z-direction in Figure 2, left) and perpendicular to it (X-direction in Figure 2, left). The sensor was placed in the middle of the thin-walled area and was repositioned after every measurement to cover the whole height of the sample. The Barkhausen measurement parameters are shown in Table 3.

The evaluation of the Barkhausen signal focuses on the RMS value because it has the lowest statistical error.

### 2.3. Stress Measurement

The residual stress analysis was performed using a DR45 diffractometer from Stresstech GmbH with Cr-Kα-radiation (wavelength = 0.2291 nm). The device was used in the modified *χ*-mode according to DIN EN 15305 [22]. The beam was masked using a collimator with a nominal diameter of 3 mm. The ψ-range extended −45° to +45° divided into six tilt angles for the positive and negative directions, which were chosen to be equidistant in $sin^{2}\psi$. To obtain information on the orientation of the stress state, measurements for ϕ= 0° and 90° were made. Here, the 0° measurements coincide with the build direction. For the stress evaluation, the $sin^{2}\psi$ method was used with a cross-correlation peak fit. The depth profiles were recorded by electrochemical polishing after the stress measurement for each depth step, allowing material removal without inducing new residual stresses. The steps were chosen between 10 μm close to the surface and 50 μm for deeper measurement points.

### 2.4. Porosity, Hardness and Wall Thickness

For the porosity measurement, polished samples were analyzed using an optical microscope in reflection mode. The evaluation was performed using a grayscale analysis. The same polished samples were used for hardness mappings using a Qness Q10 A+ automatic hardness testing device. These measurements were performed with HV0.1 and a spacing of 0.5 mm between each point. The thickness of the wall was measured using an outside micrometer with a flat measuring face. Three measurements were performed for each sample and averaged.

## 3. Results

As shown above, microstructural and mechanical properties have an influence on the MBN of ferromagnetic materials. In the following subsections, the different properties are characterized using established methods. Moreover, the results of the MBN measurements are shown. Since the surface roughness measured with a confocal microscope shows no dependence of the thickness of the samples or the position, the roughness is not discussed later.

### 3.1. Specimen Characterization

**Figure 4:**
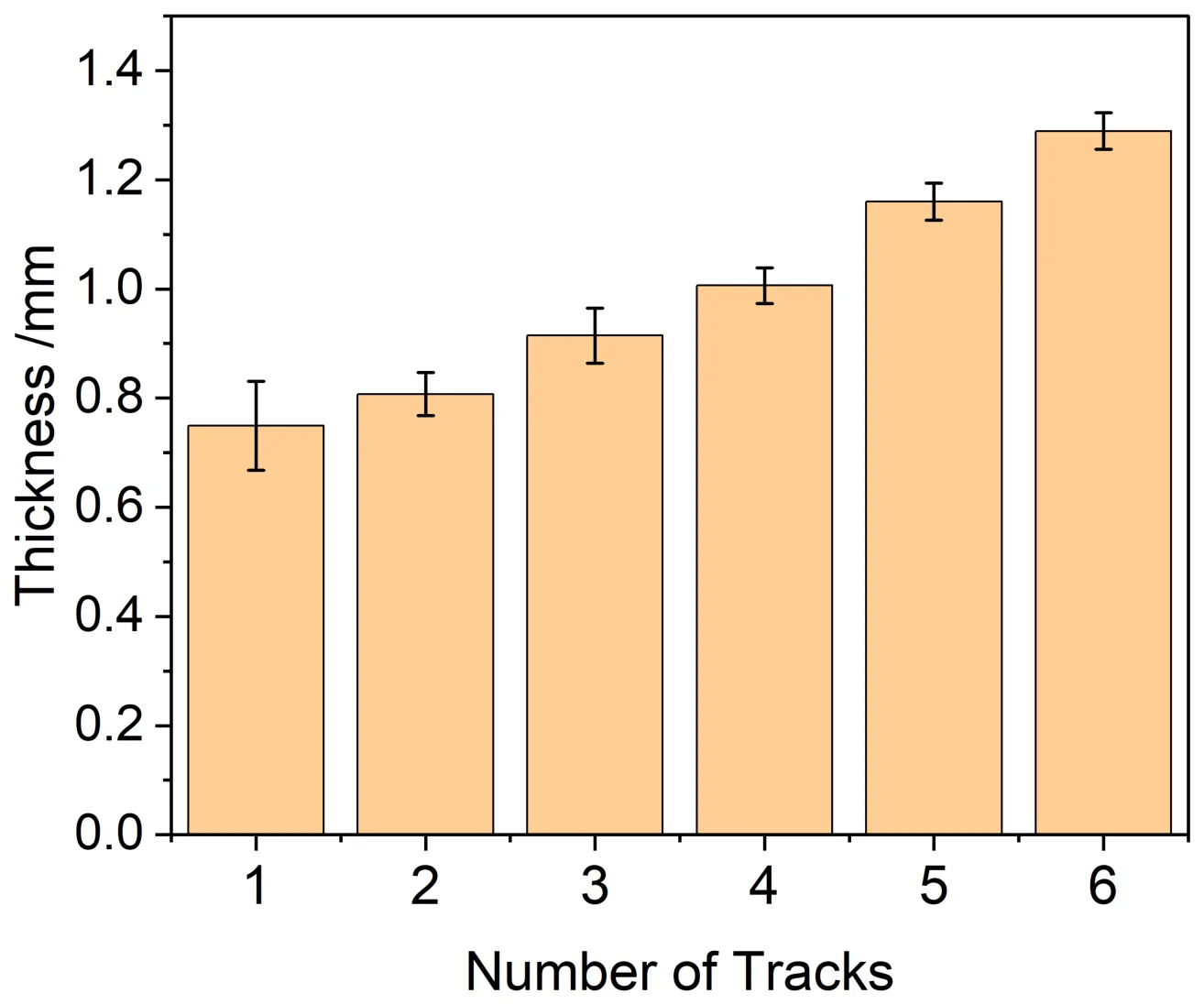
Dependence of the wall thickness on the number of scan tracks.

Figure 4 shows the relationship between wall thickness in thin-walled areas and the number of scan tracks. As expected, there is a linear correlation. The average increase in thickness per scan line is 110 µm, which is slightly lower than the 140 µm hatch distance between laser paths.

**Figure 5:**
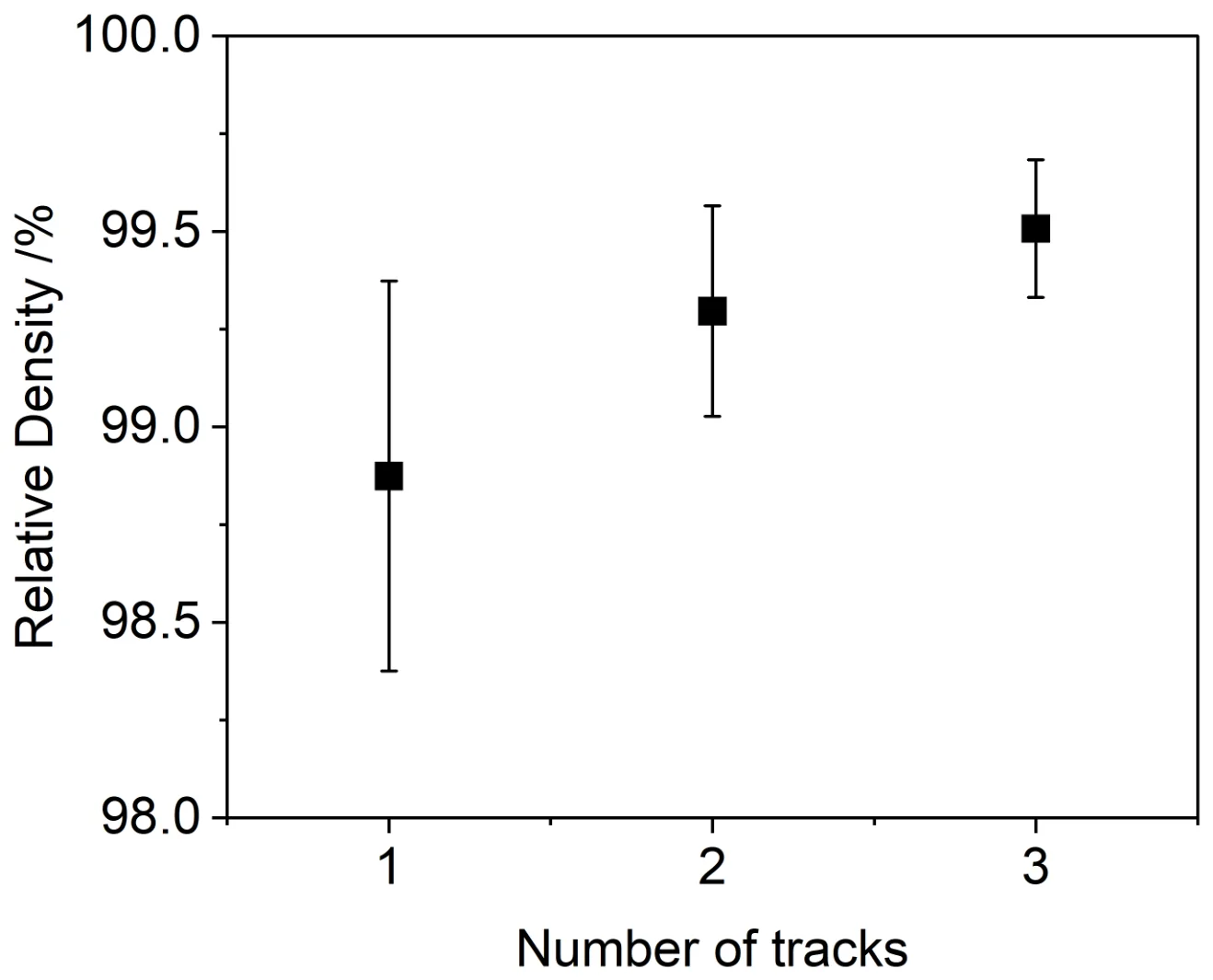
Relative density as a function of the number of scan tracks.

**Figure 6:**
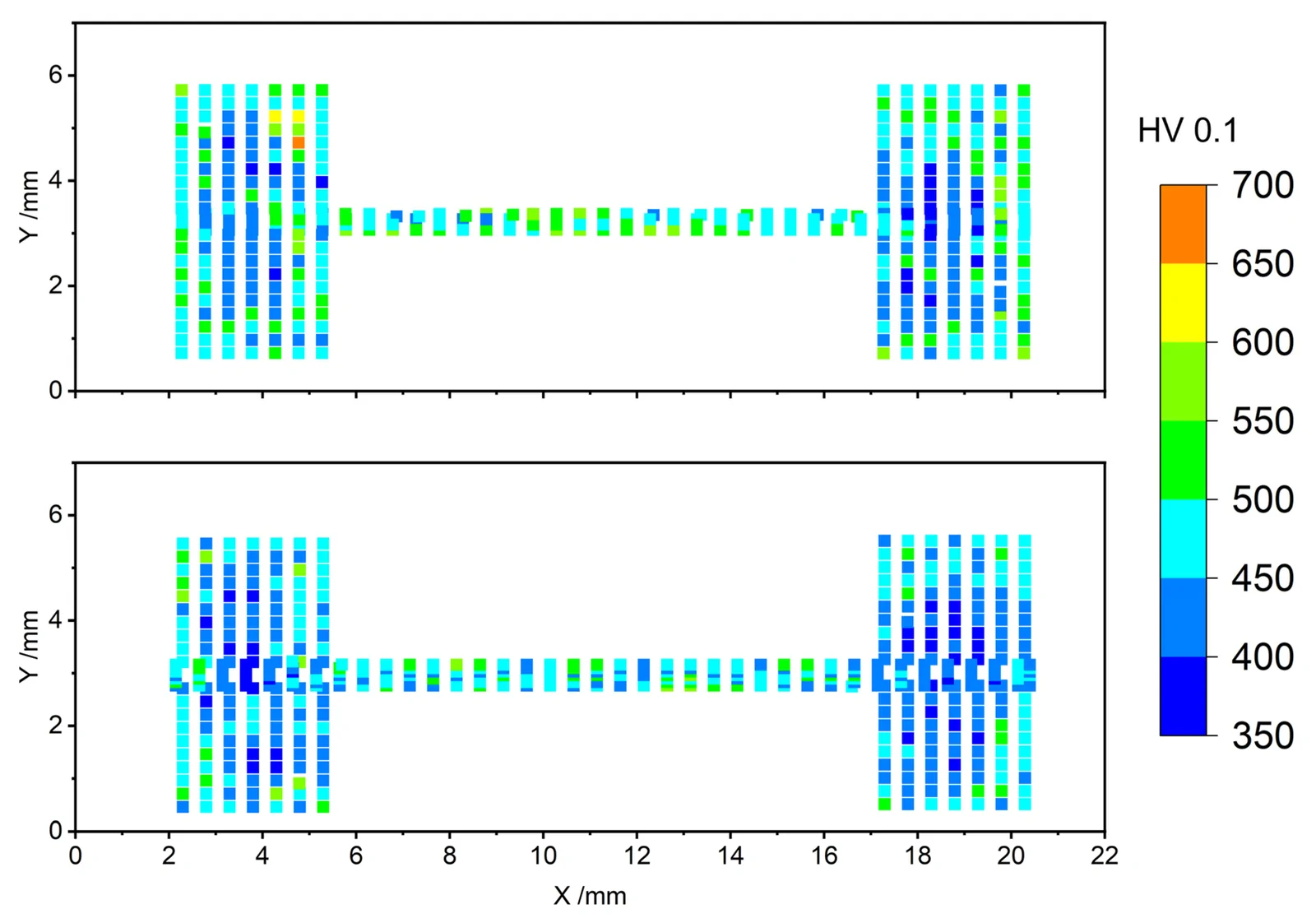
Hardness mapping on the XY cut of samples with two (**top** panel) and three (**lower** panel) scan lines.

For the determination of the porosity, the thin-walled area and the thicker support structure were measured in the XY and YZ cuts of four samples per thickness using a gray-scale analysis. In general, the relative density of the thicker support frame is 99.7%, which is higher than the average density of 99.3% of the thin walls. Figure 5 presents the density of samples with a lower wall thickness. The Z-direction represents the build direction. The relative density increases with the number of laser tracks. However, the changes are within the standard deviation. Exemplary hardness maps recorded on the XY cut of samples with two and three laser tracks in the thin-walled area are shown in Figure 6. The x- and y-axes have been scaled differently to facilitate the localization of the hardness values. The thin-walled areas show a higher average hardness (488 HV0.1) compared to the thicker support structures (470 HV0.1). In the support structure, the measurement points close to the surface show a higher hardness, which is comparable to the hardness of the thin wall. There are no significant differences in the average hardness of the thin-walled areas between samples with a different number of tracks.

### 3.2. Microstructural Analysis

Due to its complex thermal history, AISI 4140 manufactured by PBF-LB exhibits a unique microstructure that influences its mechanical properties. Since the thermal history also depends on the geometry (e.g., the wall thickness), the microstructure is also expected to depend on the geometry.

**Figure 7:**
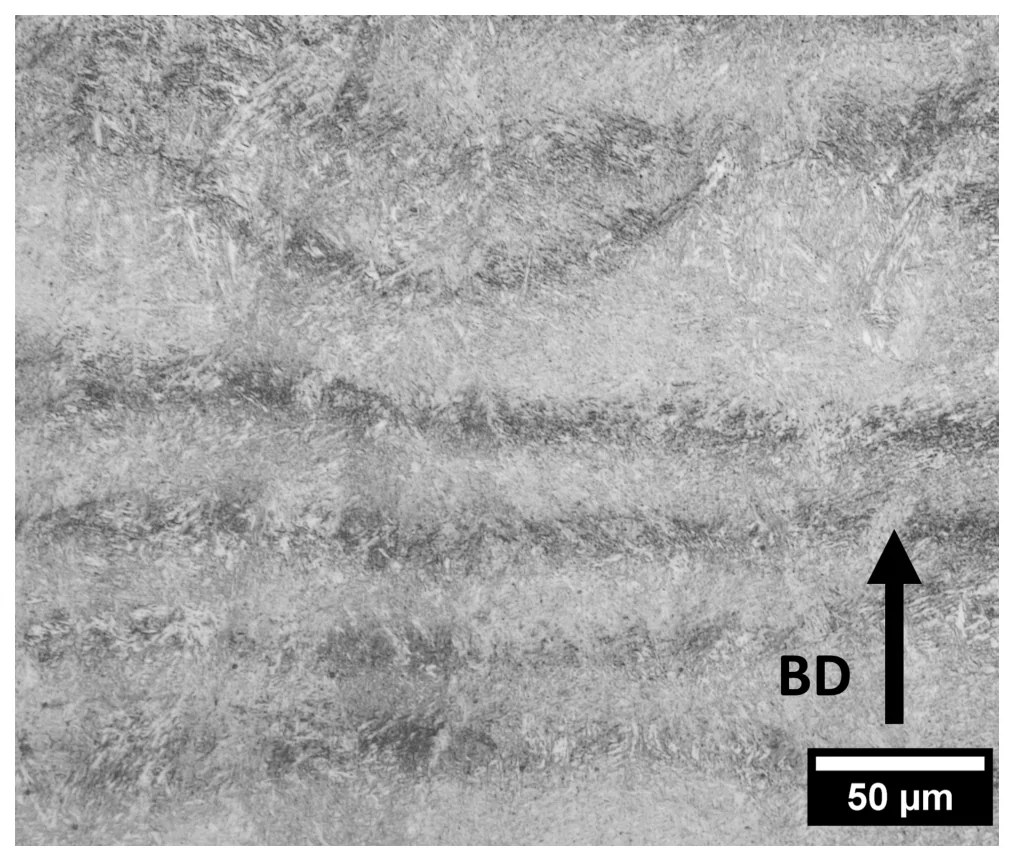
Light microscopy images captured parallel to the build direction (BD).

**Figure 8:**
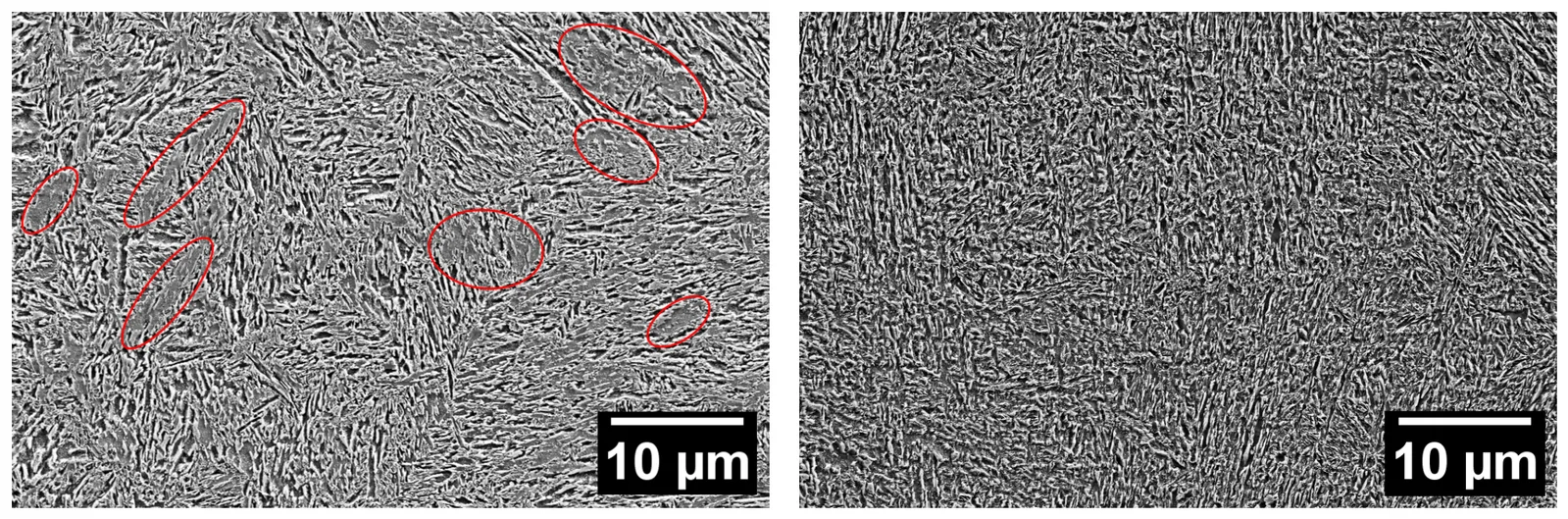
SEM images from the support structure (**left**) and the thin-walled area (**right**).

**Figure 9:**
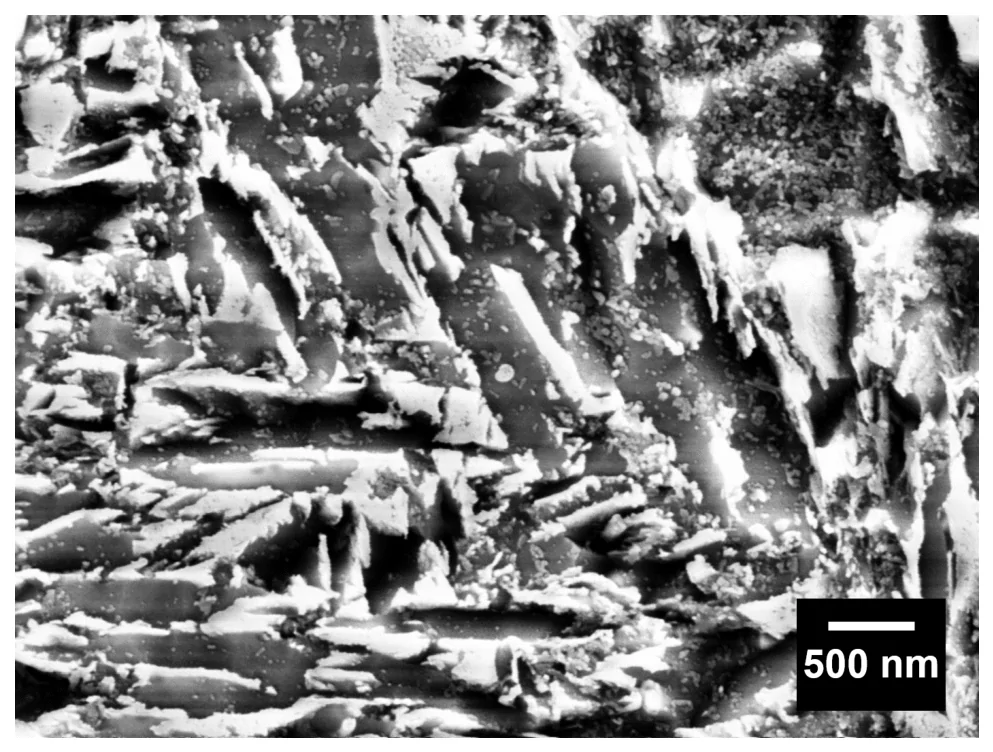
SEM images of the support structure.

The micrographs shown in Figure 7 reveal a lamellar microstructure with a cellular morphology along the build direction (BD), which is consistent with the martensitic microstructure expected for PBF-LB manufactured AISI 4140. These cells are formed by the heat-affected zones generated during the melting process as a result of tempering effects. Figure 8 shows SEM images of the frame structure and the thin-walled regions. In the massive frame structure, distinct areas are present between the martensite lamellae. These regions are highlighted in red, and a higher-magnification image is shown in Figure 9. The higher-magnification image in Figure 9 indicates that these regions contain a higher density of fine precipitates, which are attributed to cementite based on their morphology and the reported tempering behavior of AISI 4140.

### 3.3. Magnetic Barkhausen Noise

**Figure 10:**
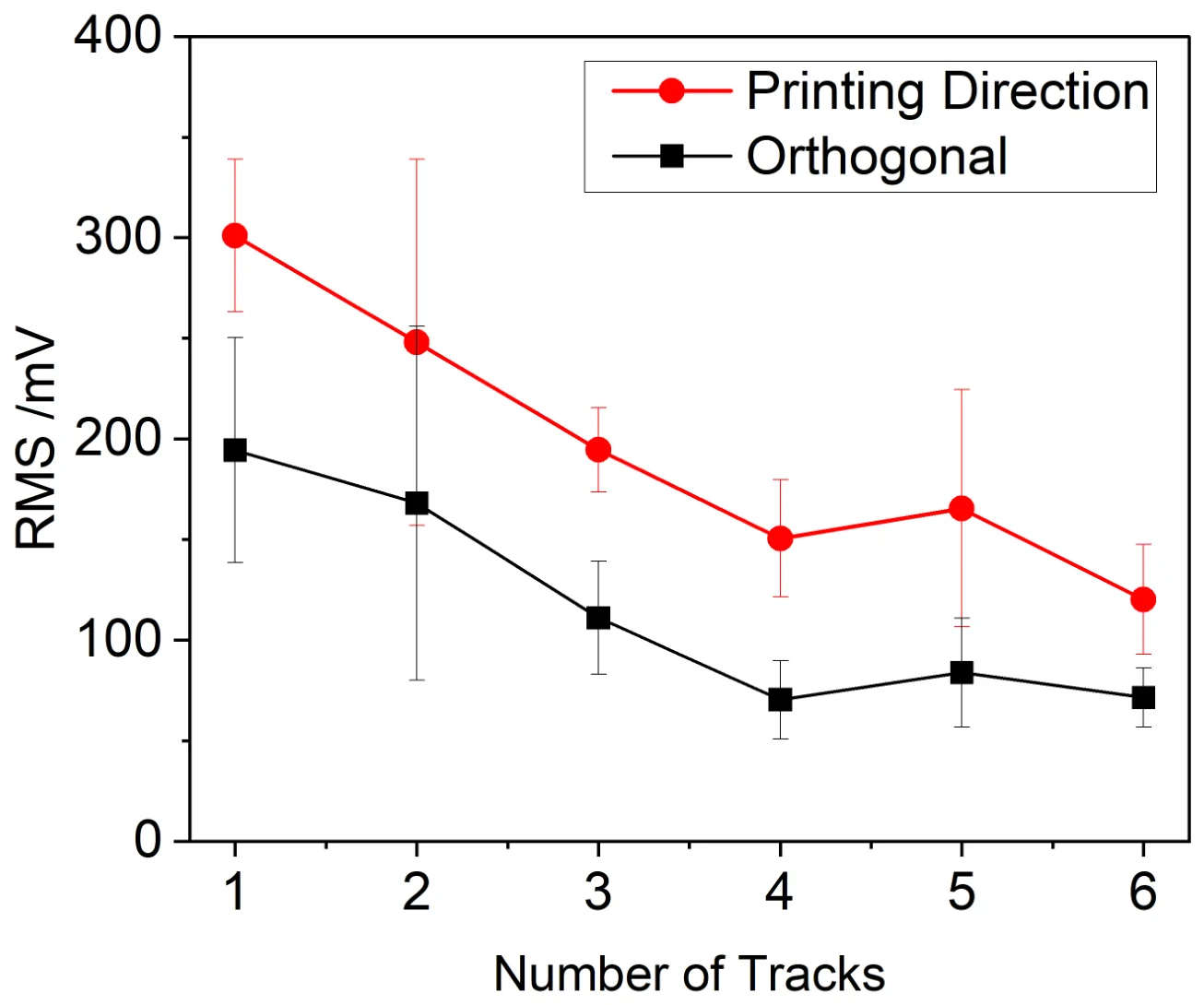
RMS dependent on the number of tracks for thin-walled samples.

**Figure 11:**
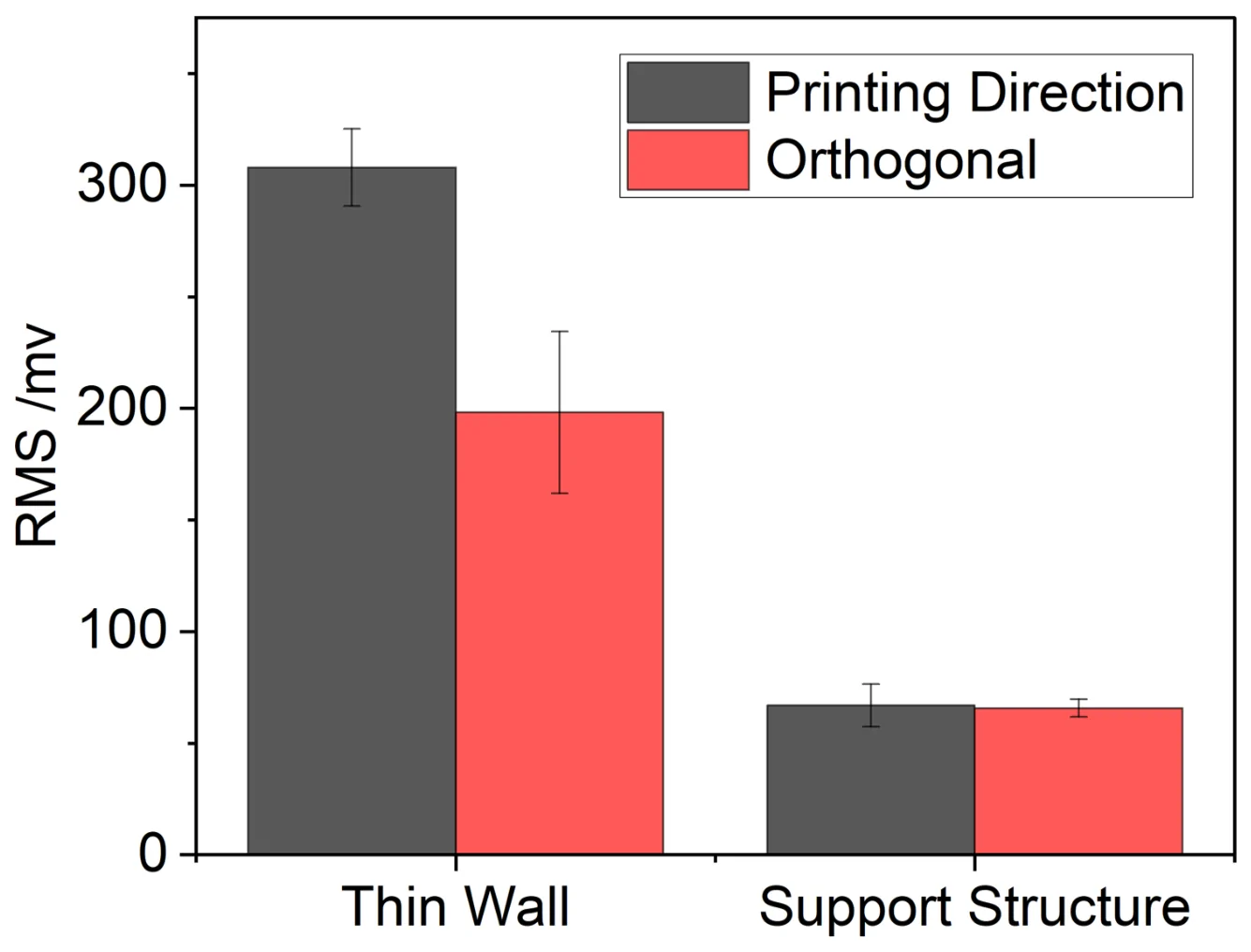
RMS compared for a thin sample with one laser path and the support structure.

Figure 10 shows the dependence of the RMS value on the number of scan tracks in thin-walled areas. The RMS value decreases exponentially with an increasing number of scan tracks. The RMS measured in the build direction is 30–40 mV lower than the RMS measured orthogonally to the build direction. For the measurements along the build direction, the thin-walled areas exhibit an RMS value four to five times higher than that of the support structures, as shown in Figure 11. For orthogonal measurements, this difference is smaller because the support structure does not show a significant directional dependence of the RMS value.

### 3.4. Residual Stresses Results

**Figure 12:**
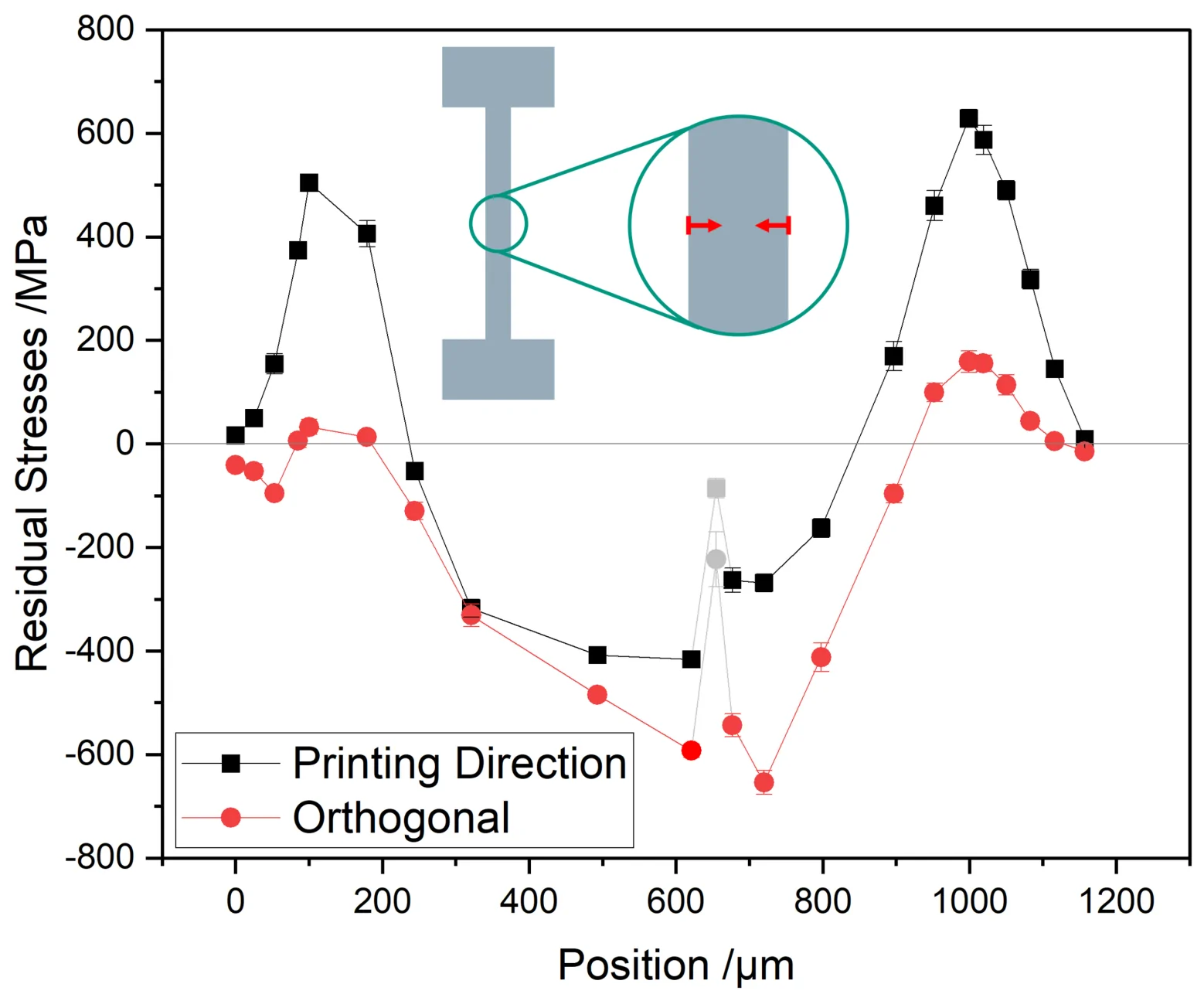
Residual stress profile through the entire sample with 6 laser tracks.

A representative residual stress depth profile that spans the entire thickness of the sample is shown in Figure 12. Electrochemical polishing was performed from both sides. At a depth of 650 µm, the breakthrough appeared in the remaining sample. This data point is marked in gray and excluded from the analysis. The residual stress profile is symmetrical, with a stress-free state on the surface, tensile stresses reaching a maximum depth of 200–250 µm and compressive stresses beyond this depth. The maximum tensile stresses are 630 MPa and the maximum compressive stresses are 650 MPa. The symmetry of the stress profile indicates that the removal of material does not lead to significant relaxation effects. This may be attributed to the presence of the support structure.

## 4. Discussion

### 4.1. Barkhausen Noise and Wall Thickness

The decrease in the MBN signal with increasing wall thickness shown in Figure 10 can be attributed to the magnetic penetration depth of the selected parameter set. The information depth is calculated according to Equation (1). It describes the depth, where the decrease factor is $e^{-1}$.

(1) $$\delta =\frac{1}{\sqrt{\pi \mu \sigma f}}$$

with

(2) $$\mu =\mu_{r}\cdot \mu_{0}$$

The measurements in this study were performed using a magnetization frequency *f* of 80 Hz. The electrical conductivity was calculated from the specific electrical resistivity of $\rho =0.19\;\frac{\Omega \;{\mathrm{mm}}^{2}}{m}$ reported in the manufacturer’s datasheet for AISI 4140 steel, resulting in $\sigma =1/\rho =5.263\cdot 10^{6}\;\frac{1}{\Omega \;m}$. Together with the magnetic field constant $\mu_{0}=1.2566\cdot 10^{-6}$, Vs/Am and a relative permeability $\mu_{r}$ ranging from 300 to 1000, the calculated penetration depth ranges from 780 to 1420 μm. The relative permeability $\mu_{r}$ was assumed to range from 300 to 1000, covering experimentally determined permeability values reported for AISI 4140 steel [23]. The relatively broad range was selected to account for the strong influence of the heat treatment condition on the magnetic properties, which may vary locally within additively manufactured parts due to the localized thermal history of the AM process. The magnetization frequency of 80 Hz was used for this estimation because it corresponds to the magnetization frequency applied during the MBN measurements. Since the magnetic penetration depth decreases with increasing frequency, the use of 80 Hz provides the maximum penetration depth within this frequency-based estimation. The MBN signal itself is broadband and contains higher-frequency components, which consequently exhibit lower penetration depths. Thus, the calculated range should be regarded as an estimate of the maximum characteristic penetration depth rather than as a sharply defined measurement depth. The magnetization frequency was chosen such that the calculated penetration depth is comparable to the thickness of the thinnest samples. Therefore, the magnetic field could be concentrated because it has no material to expand, as Equation (1) gives the depth where the decrease factor of $e^{-1}$ is reached. Due to the concentration of the magnetic field, increased domain wall movement occurs near the measurement coil, resulting in a higher MBN signal. In [24], Garstka showed similar behavior for conventional manufactured materials. With a decreasing thickness of the sample, he measured an increasing Barkhausen signal. In this study larger wall thicknesses and different Barkhausen parameters, which were optimized for the measurement on thicker samples, were investigated.

### 4.2. Comparison and Interpretation of the Residual Stress Profile

The residual stress profile shown in Figure 12 aligns with the findings of the literature on various materials produced via PBF-LB. The stress-free state at the surface is likely due to the outer powder layer being attached to the bulk material through sintering effects, while the powder itself remains stress-free. The XRD measurement has an information depth of 7–10 µm, which is lower than the average particle size of the powder. The tensile stresses underneath and the compressive stresses in the middle of the wall show a similar behavior, as already shown for the surface area of bulk samples with simulations [25] and with an experimental approach [6]. Unlike in the referenced studies, where a contour-hatch strategy was used, the parts examined in this study were only fabricated with contour tracks in the thin areas. Consequently, this residual stress profile does not originate in the contour-hatch strategy. Moreover, in the referenced studies, the residual stresses were measured by a different method. There, the residual stresses were calculated by measuring the deformation of the cutting plane. The only deviation between the measurements with the two measurement principles is observable at the surface. Following that, the resolution of the measurement of the deformation of the cutting plane is much lower, the stress-free state of the surface cannot be resolved.

The symmetric behavior of the depth profile indicates that the support frame prevents stress redistribution, although the amount of material removed is significant compared to the remaining material.

### 4.3. Hardness, Microstructure and Density

The hardness mapping shows a higher average hardness in the thin-walled area and in the contour tracks of the support structure compared to the inner part of the support structure. The difference between the support structure including the contour tracks and the thin walls is 18 HV0.1. This could be due to the longer average scan vector line in this area. Longer scan vectors lead to a longer cooling phase in between two melting cycles. This effect is amplified by the scanning strategy of the sample. One contour path is melted for the entire part, before the second one gets melted. As a result, it takes a long time for one point to get re-melted and there are fewer remelting cycles, because there are not as many adjacent scan vectors. Therefore, these parts go through fewer reheating steps. Each reheating step causes tempering effects. With less tempering, the microstructure has a higher martensite content, leading to higher hardness. This also is consistent with the differences in the microstructure shown in the SEM (Figure 8). In the thicker-walled support structure, larger areas with fine cementite precipitates are visible. As [26] showed, these precipitates form during a tempering process and also lead to lower hardness and tensile strength.

On the other hand, areas close to the surface of the part exhibit different heat-transfer conditions than areas within the hatch region. Since one side is insulated by the surrounding powder, which has low thermal conductivity, the cooling rate is expected to decrease. This would lead to a lower temperature difference within the melting cycle and a lower martensite content in the thinner parts. However, in this case, the effect of the longer scan vectors appears to be dominant, since the higher hardness is recorded.

### 4.4. Influence of the Microstructure, Hardness and Residual Stresses on the MBN

**Figure 13:**
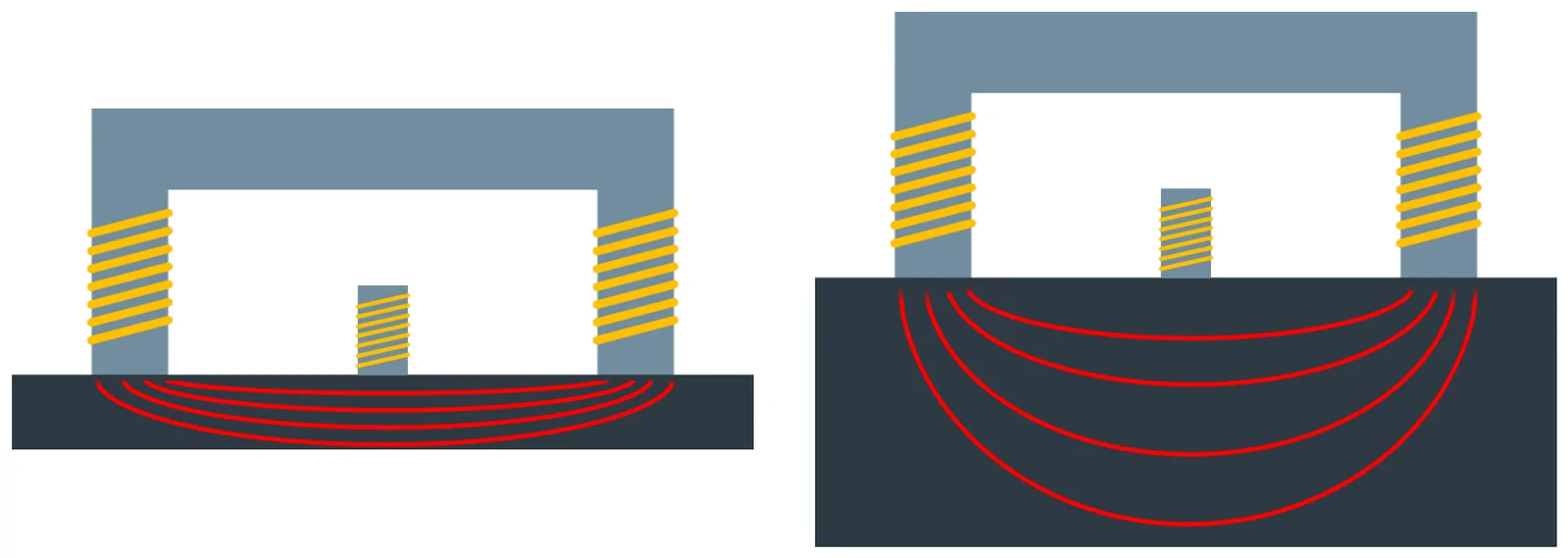
Magnetization of a thin (**left**) and a thick sample (**right**).

As shown in [27] a higher hardness leads to a lower MBN because a softer material state also has fewer defects, resulting in fewer pinning events and easier movement of domain walls. As a consequence, the MBN is higher. In this study, a higher MBN signal was detected in the harder regions of the sample, which is not consistent with the literature. Therefore, other effects must compensate for the influence of the hardness. Because the magnetic field induced in the material decreases exponentially, the highest magnetic field density is present in the near surface area, where tensile stresses are present. These tensile stresses result in a higher Barkhausen signal. But as shown before, the residual stress profile and hence stress-related changes in the MBN are not affected by the thickness of the samples. Consequently, the primary influencing factor appears to be geometric. In addition to the influence of hardness, the different tempering conditions also affect the precipitation of carbides. As discussed above, a higher density of fine cementite precipitates was observed in the thicker-walled support structure. These precipitates can act as pinning centers for magnetic domain walls and thereby influence the MBN response. Thus, the higher carbide density may contribute to the lower MBN signal observed in the support structure, despite its lower hardness. However, the available data do not allow the individual contributions of carbide precipitation and geometry to be quantitatively separated. The observed geometric effect could be explained by a concentration of the magnetic field. By applying the same magnetization energy to a different amount of material, the density of the magnetic field is higher in the thinner sample. This effect is visualized in Figure 13.

### 4.5. Anisotropy

The observed anisotropy can primarily be attributed to the residual stress state. Anisotropic tensile stresses, as they are present close to the surface in build direction (see Figure 12), lead to a higher MBN (see Figure 11). In [28] the authors have shown for conventionally manufactured parts that tensile stresses increase the MBN. This study investigated the effects on rolled steel sheets. Since similar effects are shown for additively manufactured parts, the findings appear to be comparable.

### 4.6. Transferability to Other Materials

The observed thickness-dependent MBN response is not expected to be specific to AISI 4140, but may also occur in other ferromagnetic steels used for additive manufacturing. This is supported by the findings of Garstka [24], who reported a similar increase in the Barkhausen signal with decreasing sample thickness for conventionally manufactured S235JGR2 steel. The relevant wall thickness range, however, depends on the magnetic penetration depth, which is influenced by the electrical conductivity, magnetic permeability, excitation frequency, and microstructural state of the material. Therefore, a similar geometry effect may be expected for other ferromagnetic AM steels, while the characteristic thickness range and magnitude of the effect may differ.

For non-ferromagnetic metallic materials, the magnetic response differs fundamentally from that of ferromagnetic steels. Consequently, the specific MBN-based geometry effect investigated in this study cannot be directly transferred to such materials. Nevertheless, analogous geometry-dependent effects may occur when other NDT techniques are applied. For example, eddy-current-based methods also exhibit a frequency-dependent penetration depth, such that the relationship between penetration depth and component thickness can influence the measured signal. The relevant thickness range and physical mechanism would, however, depend on the respective NDT technique and material properties.

## 5. Conclusions

In this study, Magnetic Barkhausen Noise (MBN) measurement was successfully applied to additively manufactured thin-walled geometries made of AISI 4140. When the thickness of a measured volume is smaller than the penetration depth of the magnetization, the MBN signal becomes amplified. It is important to note that the magnetic field intensity decreases exponentially with depth, meaning that a larger volume than the theoretically calculated penetration depth must be considered. Moreover, the influence of the stress state has been shown by the detection of anisotropy in the Barkhausen signal and stresses close to the surface.

The main findings of this study are:

- Tensile residual stresses, which are characteristic of AM parts, enhance the MBN signal. Therefore, MBN can be used to assess tensile residual stresses.
- Anisotropic effects in additively manufactured parts, linked to residual stress distribution, can be detected via MBN.
- An increase in laser path length leads to higher hardness in AM parts. However, this difference was not reflected in the MBN measurements because the expected effect was compensated by the effect of the geometry.
- Part geometry significantly influences the MBN response, particularly when the thickness of the material is less than the penetration depth.

Further investigations should focus on varying the microstructural state to assess whether additional microstructural characteristics influence MBN. Additionally, modifying measurement parameters may help minimize geometric influences and provide more detailed insights into the stress state.

## Figures and Tables

**Table 1:** Chemical composition (wt.%). Fe balance.

	C	Si	Mn	Cr	Mo
Standard [21]	0.38–0.45	0.1–0.4	0.6–0.9	0.9–1.2	0.15–0.3
Measured	0.39	0.34	0.76	1.09	0.25

**Table 2:** Manufacturing parameters.

Laser Power	Spot Size	Hatch Distance	Scan Speed	Layer Height
[W]	[µm]	[µm]	[mm/s]	[µm]
250	80	140	300	30

**Table 3:** Barkhausen parameters.

Number of Bursts	Magnetization Frequency	Magnetization Voltage	Waveform
[-]	[Hz]	[V]	[-]
10	80	5	Sine

## Data Availability

The data presented in this study are available from the corresponding author upon reasonable request. Public deposition of the raw datasets is not feasible because they comprise extensive experimental measurement data generated using specialized characterization equipment and require dedicated software and documentation for proper interpretation. During the preparation of this work, the authors used Writefull and ChatGPT-5 to improve the readability of the manuscript. After using these tools, the authors reviewed and edited the content as needed and take full responsibility for the content of the publication.

## Abbreviations

The following abbreviations are used in this manuscript:

MBN	Magnetic Barkhausen Noise
PBF-LB	Laser beam powder bed fusion
AM	Additive manufacturing
NDT	Non-destructive testing
FWHM	Full width at half maximum
RMS	Root mean square
BD	Build direction
